# Extravasation of Intravenous Iron: Clinical Features and Therapeutic Considerations

**DOI:** 10.1155/crh/5531805

**Published:** 2026-01-29

**Authors:** Bilge Ada Özcan, Fadime Yılmaz, Abidin Gündoğdu, Gokhan Tazegul, Zekaver Odabaşı

**Affiliations:** ^1^ Department of Internal Medicine, Marmara University Pendik Research and Training Hospital, İstanbul, Turkey; ^2^ Department of Internal Medicine, Division of General Internal Medicine, Faculty of Medicine, Marmara University, İstanbul, Turkey, marmara.edu.tr

**Keywords:** anemia, extravasation of diagnostic and therapeutic materials, ferric carboxymaltose, iron, iron deficiency anemia

## Abstract

Iron deficiency anemia is a common condition that can be effectively treated with intravenous iron supplementation; however, extravasation during administration represents a relevant adverse effect. This case series presents three patients with varying underlying conditions who experienced iron extravasation following ferric carboxymaltose infusion. The extent and timing of skin discoloration varied, with some patients developing immediate discoloration and others noticing them several days postinfusion. Despite extravasation, two of the three patients demonstrated improvement in their anemia without additional treatment during follow‐up. This series highlights the need for preventive strategies, such as careful infusion techniques, patient education, and prompt action when extravasation occurs. Equally important, accurate documentation and continuing education for healthcare professionals are essential to ensure consistent recognition and management. Further studies are required to clarify the impact of extravasation on therapeutic efficacy and to optimize the balance between treatment benefits and potential risks.

## 1. Introduction

Iron deficiency and iron deficiency anemia (IDA) can arise from a range of causes, including inadequate dietary intake and impaired gastrointestinal absorption, as well as chronic medication use, ongoing blood loss, or renal insufficiency [1]. In most cases, oral iron supplementation is the preferred first‐line therapy because it is effective, safe, and inexpensive. Yet, when oral iron is not viable—because of gastrointestinal intolerance or impaired absorption, as seen in conditions such as atrophic gastritis, gastrectomy, or active gastrointestinal bleeding—intravenous (IV) iron is considered. Beyond these scenarios, IV iron has also demonstrated superior efficacy over oral therapy in evidence‐based indications, including chronic inflammation, chronic kidney disease, heart failure, and perioperative anemia [2]. Among IV iron preparations, ferric carboxymaltose is preferred for its convenience and efficacy; however, like other formulations, it carries potential adverse effects, with extravasation being a significant complication.

In this case series, we describe three cases of iron extravasation following IV ferric carboxymaltose infusion, to raise awareness of the potential risks associated with IV iron therapy and to provide practical insights into its management.

## 2. Case Reports

### 2.1. Case 1

**Figure 1:**
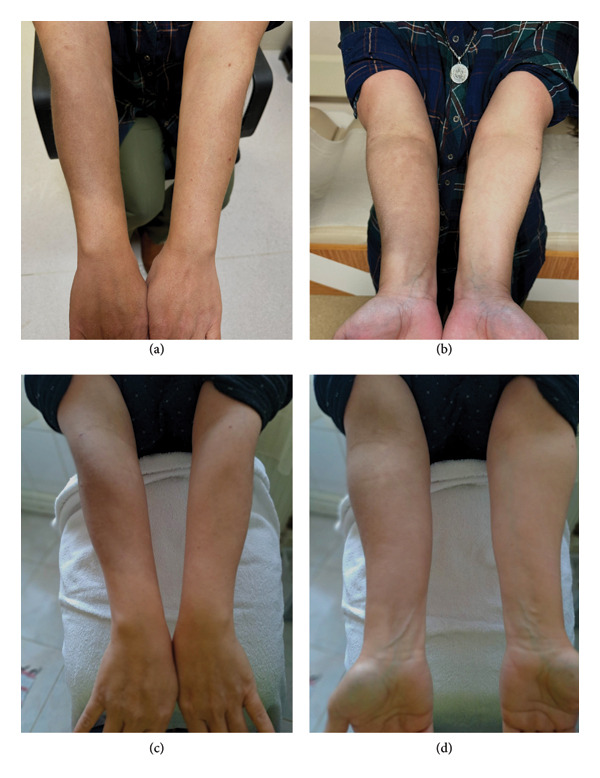
(a, b) Brown discoloration and swelling around the right antecubital vascular access site, spreading to the flexor and extensor surfaces of the forearm, immediately after ferric carboxymaltose extravasation. (c, d) Follow‐up images at 3 months showing diminished skin discoloration.

A 47‐year‐old woman with hypothyroidism came to the clinic complaining of weakness, fatigue, and hair loss. She was on a daily dose of 125 mcg levothyroxine. Despite 2 years of oral iron therapy, her anemia persisted. Laboratory results showed hemoglobin (Hgb) of 10.5 g/dL, mean corpuscular volume (MCV) of 66.7 fL, ferritin of 2 μg/L, and transferrin saturation of 4%. Thyroid function tests were within normal limits. She tested positive for anti‐parietal antibodies. She received 1000 mg IV ferric carboxymaltose. During infusion, brown discoloration and swelling developed around the right antecubital vascular access site, spreading to the flexor and extensor surfaces of the forearm, without erythema. The infusion was stopped, and cold compresses were applied (Figures 1(a) and 1(b)). Laser treatment was recommended to the patient; however, she declined due to financial concerns. During the follow‐up visit 3 months later, it was noted that the color change caused by extravasation had diminished (Figures 1(c) and 1(d)). The patient had a Hgb level of 14.6 g/dL and a ferritin level of 19 μg/L.

### 2.2. Case 2

**Figure 2:**
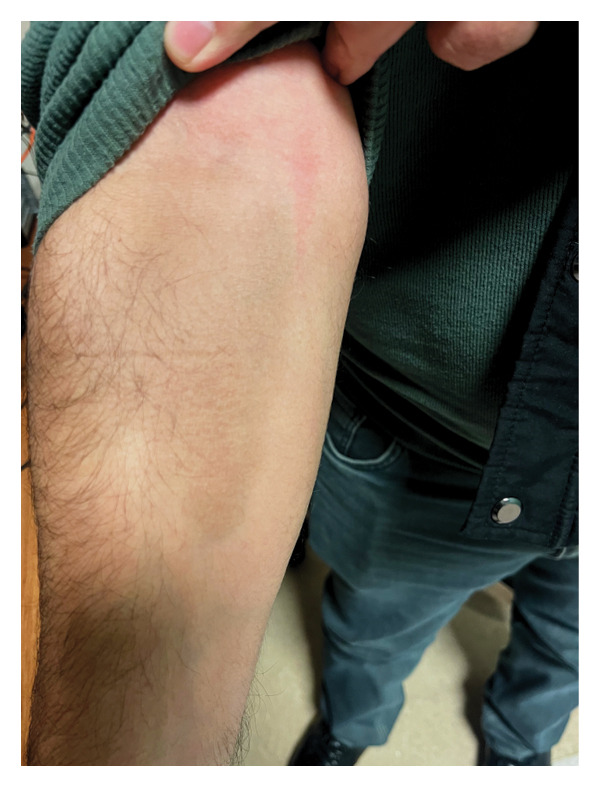
Brown discoloration on the flexor side of the right forearm at the vascular access site, observed several days after ferric carboxymaltose infusion, without erythema or edema.

A 47‐year‐old man presented to the clinic reporting of weakness, fatigue, and palpitations. His past history included anemia due to intermittent hemorrhoidal bleeding, previously managed with oral iron supplements. Upon examination, the patient exhibited conjunctival pallor, and a rectal exam revealed the presence of hemorrhoidal packs. Laboratory tests showed the following results: Hgb of 9.7 g/dL, MCV of 65.7 fL, and ferritin of 5.5 μg/L. He was experiencing active hemorrhoidal bleeding and had IDA that did not respond to long‐term oral iron therapy. He was treated with an uneventful infusion of 1000 mg of IV ferric carboxymaltose. Several days later, he developed brown discoloration on the flexor side of the right forearm at the vascular access site, without erythema or edema (Figure 2). Laser treatment was recommended; however, he declined it as he did not perceive a cosmetic need. At 3 months, his anemia had resolved (Hgb of 13.9 g/dL), but the skin discoloration persisted.

### 2.3. Case 3

**Figure 3:**
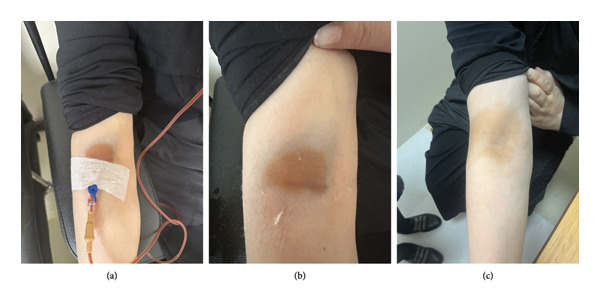
(a, b) Orange‐brown discoloration with an ecchymotic halo at the right antecubital skin, without edema or erythema, during ferric carboxymaltose infusion. (c) One week follow‐up image showing resolved ecchymotic halo but persistent discoloration spreading to the lateral surface of the arm.

A 41‐year‐old woman was found to have IDA during her preoperative evaluation for excision of a lower extremity mass, leading to referral for further management. Her laboratory results revealed Hgb of 9.9 g/dL, MCV of 71.5 fL, transferrin saturation of 3%, and ferritin of 7 μg/L. Given that her IDA was identified in the preoperative setting, IV iron therapy was recommended. She received 1000 mg of IV ferric carboxymaltose. During the infusion, orange‐brown discoloration with an ecchymotic halo appeared at the right antecubital skin, without edema or erythema (Figures 3(a) and 3(b)). The infusion was stopped, and cold compresses were applied. One week later, the ecchymotic halo resolved, but the discoloration persisted and spread to the lateral surface of the arm (Figure 3(c)). The patient was informed and remains under follow‐up.

## 3. Discussion

IV iron extravasation, though relatively uncommon, is an important clinical complication. The incidence with ferric carboxymaltose is estimated at around 1.6% [3], and pharmacovigilance data further confirm its relevance: As of May 2025, the US Food and Drug Administration’s FAERS system has documented over 250 cases, while the European Medicines Agency’s EudraVigilance database has reported more than 500 instances of skin hyperpigmentation, mostly classified as nonserious adverse events. The dermatologic outcome, however, is often unfavorable. Cutaneous hyperpigmentation caused by hemosiderin deposits tends to persist, leaving a lasting cosmetic and psychological burden [4]. Previously published case series and systematic reviews consistently report that spontaneous resolution is rare; affected patients require active therapeutic intervention to achieve cosmetic improvement [5, 6]. Together, this measurable frequency and poor dermatologic outcome underscore the clinical importance of recognizing and managing extravasation in routine practice, a relevance further illustrated by the three cases we report here.

Beyond its frequency and prognosis, understanding the factors that increase the likelihood of extravasation is equally critical. These can be broadly categorized into patient‐related, operator‐related, and product‐related risk factors. Patient‐related risks include structural venous changes that impair cannulation, comorbid vascular diseases, concurrent anticoagulation, and advanced age. Another consideration is that darker skin tones may delay early recognition of extravasation. Operator‐related risks involve limited staff experience, repeated cannulation attempts, inadequate catheter fixation, and excessive infusion pressure. Product‐related contributors, such as medication properties and infusion characteristics, also play a role [7]. Our cases demonstrate that extravasation may occur regardless of indication, and in two patients with anticoagulation or vascular comorbidities, patient‐related factors appeared to contribute to extravasation.

Recognizing these risks is only the first step; effective risk mitigation strategies are equally important. Published protocols and consensus guidelines outline several preventive measures [8, 9]. Patient education and informed consent regarding the possibility of permanent skin staining are essential. Prior to infusion, clinicians should document vital signs, carefully select the infusion site, and flush the cannula with normal saline to ensure patency. During infusion, a slower initial rate, regular inspection of the cannula site, and clear instructions to patients to report pain or discoloration are recommended. If extravasation is suspected, the infusion must be stopped immediately. After infusion, structured patient monitoring and accurate documentation of any adverse events are essential. Both staff and patients share responsibility in this phase: Staff should remain alert to early signs of extravasation or delayed cutaneous changes and record them appropriately, while patients should be encouraged to observe their infusion site and promptly report any new changes to the infusion site [8, 9]. Recent clinical observations also suggest that applying compression immediately after infusion may reduce the extent of cutaneous siderosis, offering a simple adjunct to standard preventive protocols [10]. In our series, extravasation was observed even under recommended practice, highlighting the importance of preventive pathways that combine technical measures with systematic education and shared vigilance. However, when extravasation does occur, clinicians should weigh available treatment options to minimize clinical consequences and address patient concerns.

Although various topical approaches—such as azelaic acid, thioglycolic acid, lymphatic drainage, depigmenting creams (e.g., hydroquinone and retinoids), chemical peels, and dermabrasion—have been attempted to manage extravasation, they have consistently shown limited efficacy in achieving pigment clearance [11]. Currently, laser therapy is the only effective method for treating extravasation [12]. Recent reports and systematic reviews show that quality‐switched (QS) and picosecond (PS) lasers are the most commonly used options for cutaneous siderosis. In published series, patients typically required multiple treatment sessions, with mean clearance rates of 50%–75%, and complete resolution was achieved in approximately half of the cases. While PS lasers theoretically offer stronger photoacoustic effects, current evidence does not demonstrate clear superiority over QS devices. Side effects such as erythema or crusting are common but temporary, and overall tolerability is favorable. Nevertheless, laser therapy remains expensive and requires specialized expertise, which limits access [5, 6]. Other experimental adjuncts, such as microneedling or topical chelators, have been reported in the broader pigment literature. Still, evidence remains anecdotal in iron extravasation, and no systematic studies have been conducted. Whether multimodal strategies that integrate laser therapy with topical or procedural adjuncts remains a topic for further research.

In our series, two of three patients demonstrated resolution of IDA despite extravasation, suggesting that a proportion of the infused iron remains systemically available. This paradoxical improvement may reflect the fact that a substantial proportion of the infused iron still entered the systemic circulation, whereas extravasated iron deposited in tissue may have been partially mobilized over time by macrophages. Alternatively, the high dose administered may have ensured adequate systemic repletion despite local loss. These speculative mechanisms highlight the need for mechanistic studies to clarify the fate of extravasated iron, as its metabolism and long‐term course have not been systematically studied, and it remains unclear whether tissue deposition contributes to eventual mobilization or represents a permanent loss. Further research is warranted to elucidate whether extravasation diminishes therapeutic benefit or whether systemic iron repletion can still be achieved despite local staining.

## 4. Conclusion

This case series demonstrates the variable clinical presentations and outcomes of iron carboxymaltose extravasation. Healthcare providers should implement preventive measures, educate patients, and intervene promptly if extravasation occurs. The effect of extravasation on treatment efficacy remains uncertain; further studies are warranted to clarify long‐term outcomes. Equally important, structured documentation of adverse events and continuing education for healthcare professionals are essential to ensure early recognition, consistent management, and improved patient care.

## Author Contributions

Concept: all; design: all; data collection or processing: all; analysis or interpretation: all; literature search: all; and writing: all.

## Funding

The authors received no specific funding for this work.

## Disclosure

All authors have approved the manuscript.

## Ethics Statement

Written informed consent was obtained from all patients for publication of this case series and accompanying images.

## Conflicts of Interest

The authors declare no conflicts of interest.

## Data Availability

The data that support the findings of this study are available from the corresponding author upon reasonable request.
